# Novel Hydrocolloids Obtained from Mango (*Mangifera indica*) var. Hilaza: Chemical, Physicochemical, Techno-Functional, and Structural Characteristics

**DOI:** 10.3390/gels8060354

**Published:** 2022-06-06

**Authors:** Ronald Marsiglia-Fuentes, Somaris E. Quintana, Luis A. García Zapateiro

**Affiliations:** Research Group in Complex Fluid Engineering and Food Rheology (IFCRA), University of Cartagena, Cartagena 130015, Colombia; rmarsigliaf@unicartagena.edu.co (R.M.-F.); squintanam@unicartagena.edu.co (S.E.Q.)

**Keywords:** mango (*Mangifera indica*) var. hilaza, waste products, agroindustry, hydrocolloids, structure, functionality

## Abstract

Background: Hydrocolloids are ingredients used to improve the technological properties of products; currently, there is a growing demand from the food industry and consumers to use natural ingredients and reduce the environmental impact. Methods: This work evaluated the effect of pH on hydrocolloid extraction from the pulp, seed, and peel of mango (*Mangifera indica*) var. hilaza and their chemical, physicochemical, techno-functional, and structural properties. Results: The main component of the hydrocolloid was the carbohydrates for pulp (22.59%) and peel (24.05%), and the protein for seed (21.48%) was corroborated by NIR spectra and associated with the technological and functional properties. The solubility increases with the temperature presenting values higher than 75% at 80 °C; the swelling index was higher than 30%, while the water holding capacity was higher in samples with higher carbohydrate content (110–121%). Moreover, a higher content of total phenolic compounds (21.61 ± 0.39–51.77 ± 2.48 mg GAE/g) and antioxidant activity (≥193.82 μMol Trolox/g) was obtained. The pH of extraction changes the color parameters and microstructural properties. Conclusions: Novel ingredients from mango pulp, seed, and peel at different pH levels have technological and functional properties that are potential use in the food industry as an alternative to the development of microstructural products.

## 1. Introduction

Hydrocolloids are high molecular weight biopolymers used in the food and pharmaceutical industries due to their techno-functional properties [1]. The biopolymeric structures present a hydroxyl group which increases the hydrophilic capacity, producing viscous aqueous dispersions. The characteristics of hydrocolloids allow their use as dietary fibers, thickeners, gelling agents, emulsifiers, stabilizers, fat substitutes, clarifying agents, flocculating agents, and clouding agents [2]. In addition, hydrocolloids have applications in research and industrial areas to develop edible films, encapsulating properties, and substance crystallization inhibitors, allowing them to be used in beverages, confectionery, dairy-based products, and bakeries [3,4]. 

Different vegetable and fruit waste have been resources for the extraction of hydrocolloids using different techniques. Conventional extraction technique with hot water is commonly used for extraction; that is, Orgulloso-Batista et al. [5] obtained hydrocolloids from *Cucurbita moschata* peel with stabilizer properties; Rojas-Torres et al. [6] from butternut squash seed with stabilization and emulsification properties; Lopez-Barraza et al. [7] from *Pereskia bleo* leaves with high water holding capacity and emulsifying stability; de Andrade Vieira et al. [8] from cacti with good water and oil retention capacities; Temenouga et al. [9] obtained hydrocolloids mucilage from okra with emulsifying properties; and Rashid et al. [10] extracted flax gum from *Linum usitatissimum* L. seeds with foaming capacity, swelling index, and foaming stability. Furthermore, ultrasound-assisted extraction (UAE) as a new method has been developed to advance the extraction process [11], i.e., ultrasound-assisted extraction and vacuum cooling to obtain peach gum polysaccharide [12]; Keshani-Dokht et al. [13] extracted mucilage from *Cordia myxa* with antiradical capacity, water solubility, and water/oil holding capacity; Ezzati et al. [14] solubility, water and oil holding capacities, emulsifying capacity and emulsifying stability and Chen et al. [15] extracted polysaccharides from okra with antioxidant capacity and high solubility. Then, microwave-assisted extraction was used for the extraction of high hydrocolloid yield in a short time, i.e., Samavati [16] obtained hydrocolloids from okra pod with antioxidant activity, and Dranca et al. [17] extracted pectin from Malus domestica with film-forming properties. Similarly, hydrocolloids obtained from vegetables and fruits have been used to develop new products, such as banana, papaya, and *Citrus sinenesis* peels in the production of yogurt [18,19]; cress seed gum with chitosan nanoparticles has been used to develop active films evaluating its encapsulation capacity for the pomegranate peel extract [20]. In addition, they have been incorporated as enhancers of the physical attributes of cakes when they are partially added as egg substitutes [21]. 

The techno-functional properties of hydrocolloids depend on their solubility in the aqueous phase of food; the ability to increase the viscosity of solutions, form gels, or stabilize emulsions requires good solubility in water [22]. Different hydrocolloids have been used in the food industry, i.e., acacia, modified starch, whey protein, casein, and soybean proteins, due to their good water solubility properties [23,24]. However, some hydrocolloids are insoluble in water depending on their molecular characteristics, particularly their surface hydrophobicity, charge, and molecular weight [25]. The viscosity of hydrocolloids depends mainly on their physical entanglement (random coils) [26]. In dispersion, the individual hydrocolloid molecules can move freely and do not show thickening or gel-forming ability. However, when there is a concentrated substance, these molecules begin to contact each other; therefore, the movement of the molecules becomes restricted, facilitating the change of viscosity. The transition from free-moving molecules to an interlaced network is a thickening process. Moreover, hydrocolloid thickeners can be considered as an entanglement of network producers. In cases where interactions between polymer and polymer are not observed, thickening properties are associated with molecular weight and hydrocolloid concentration [27,28].

Mango (*Mangifera indica*) is an *Anacardiaceae* family fruit of the *Sapindales* order and grows in many parts of the world, mainly in tropical countries [1]. Each part of a mango tree can be used, such as leaves, flowers, bark, fruit, pulp, peel, and seeds, due to essential nutrients [29]. Approximately 100 mango fruit varieties are known for diversity in size, shape, color, flavor, seed size, and chemical composition, depending on the crop, weather conditions, harvest, and post-harvest treatments. [30,31,32]. In general, this fruit is considered an essential source of micronutrients, vitamins, dietary fiber, carbohydrates, proteins, fats, and phenolic compounds in the diet [33]. The main industrial products obtained are syrup, nectar, fruit puree, canned food, and chutney; then, depending on the process, different amounts of industrial byproducts such as seeds and peels could be obtained, representing 35–60% of the fruit’s total weight [1,34,35,36,37]. The objective of this study was to obtain novel hydrocolloids from all parts of mango (*Mangifera indica*) var. Hilaza and their physicochemical, chemical, techno-functional, and structural characteristics, are capable of contributing to the development of new food science additives using renewable and environmentally friendly sources.

## 2. Results and Discussion

### 2.1. Chemical and Physicochemical Properties of Hydrocolloid

**Figure 1:**
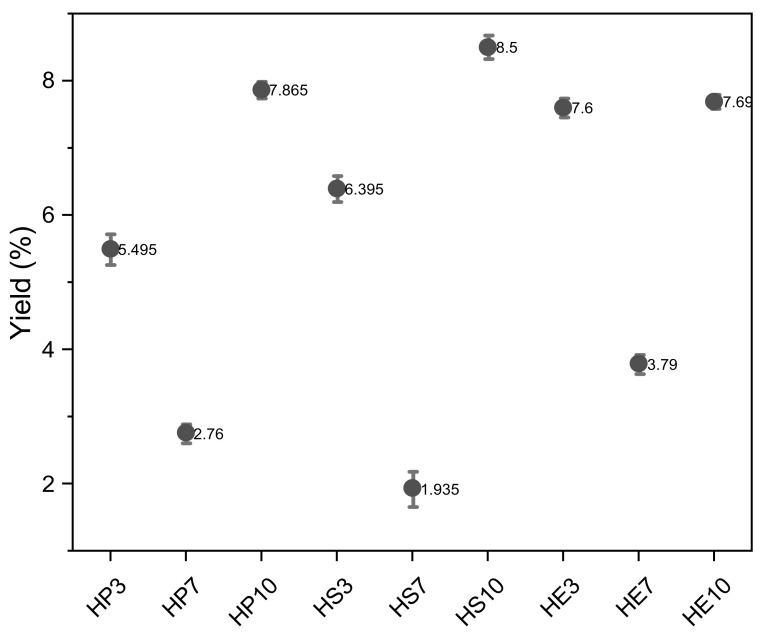
Percentage of hydrocolloid extraction from the seed, peel, and pulp of mango (*Mangifera indica*) var. hilaza. HP (pulp hydrocolloid), HS (seed hydrocolloid), and HE (peel hydrocolloid).

Hydrocolloids were extracted at pH 3, 7, and 10 from the pulp (HP3, HP7, and HP10), seed (HS3, HS7, and HS10), and peel (HE3, HE7, and HE10). The extraction yield of hydrocolloids are shown in Figure 1, indicating yields of 5.56%, 2.62%, and 7.81% for pulp; 6.34%, 1.86%, and 8.3% for seed, and 7.64%, 3.83% and 7.72% for peel, at pH 3, 7 and 10, respectively; in general the peel presents the highest yield at pH 3 and pH 7 and seed at pH 10. Then, the extraction yields at pH 3 and 10 were higher than at pH 7. At pH 10, the viscosity of the solution increased, increasing the power required to mix the slurry and thus subjecting the sample to higher shear stress; this increased shear may have resulted in a higher erosion rate and thus higher extraction [38]. Then alkaline conditions could increase the yield by hydrolyzing insoluble constituents into soluble which increases the extraction yield. This finding was in agreement with previous researchers, i.e., Balke and Diosady [38], Estévez et al. [39], and Somboonpanyakul et al. [40]. At pH 3 can improve de yield because it promotes extensive hydrolysis resulting in smaller chains that do not precipitate by adding ethanol, thus reducing yield [41]; Similar results were obtained by Colodel and Petkowic [42] and Keisandokht et al. [43]. Change in pH favors extraction of hydrocolloids due to exposure of hydrophilic groups that can interact with water and favor their extraction [17,44]. In all cases, the lowest extraction yield was obtained at pH 7. It can be deduced that the extraction of solubilized hydrocolloids at this pH did not have good yields as the denaturation capacity of polymeric systems is not adequate; the interaction of polymeric chains responds to physical, chemical, and electrical changes that are favored by changes in pH, allowing better extraction [45,46].

The chemical composition of hydrocolloids depends on their source and extraction conditions. Table 1 shows the physicochemical properties of hydrocolloids from the seed, peel, and pulp of mango (*Mangifera indica*) var. hilaza. Soluble solids present significant differences (*p* < 0.05) from mango parts, the pulp has the highest value, followed by peel and seed due to its higher sugar content [47], which does not variate with the extraction pH (*p* > 0.05). The acidity values decreased with increasing extraction pH (*p* < 0.05) and did not vary with the mango source of mango (*p* > 0.05).

Hydrocolloids have moisture values between 62.96% and 75.32%, lipid content of 0.63 and 11.15%, protein content of 0.33 and 20.93%, carbohydrates of 2.06 and 23.52%, and ash values less than 1.61%. Similar results were obtained by Cao et al. [48], Renard et al. [49], and Ma et al. [50], who extracted and characterized hydrocolloids from vegetable raw materials, such as camelina gum isolation, acacia gums (*Senegal andseyal*); and Chickpea flour as a functional ingredient in foods. Furthermore, the protein content is higher than flaxseed gum [51] compared to hydrocolloids extracted from the mango seed.

Similarly, Estévez [39] carried out a study on the influence of pH on the level of fat, protein, and ash content. The main content of the hydrocolloids was protein from seeds and carbohydrate from peel and pulp, which could be associated with the techno-functional properties of the samples. The values of the ash content were similar to those obtained by Escobar et al. (0.9–2.1 g/100 g DM) [52] and commercial guar gum (1.5%) and locust bean gum. The lipid content was lower for hydrocolloids from peel and pulps; in acid extraction (pH 3), the lipid content was lower, whereas the level in alkali-extracted gum was similar to that reported by Ibañez and Ferrero [53], while for hydrocolloids from seed were higher (>9%) associate to the higher lipid content of mango seed [54]. The protein content was higher in HS (20%) and lowered in HP and HE when the acid extraction (pH 3) resulted in a lower protein content owing to molecular hydrolysis caused by the acid. The higher protein and ash content is due to the increased damage of the sample during the extraction of hydrocolloids. HS presents the lowest carbohydrate content (<2%) and the highest value of HP, and HE has the highest value (>20%). This characteristic can positively impact the formation of emulsions due to the hydrophilic and hydrophobic attributes of the proteins, which present values higher than those obtained by Lopéz-Barraza et al. [7] based on gums extracted from *Pereskia bleo* Leaves, Gannasin et al. [46] of gums extracted from tamarind seed; in addition, the proximal composition of the hydrocolloids in terms of pH changes, presented minimal significant differences with respect to the extraction material.

Measurements of color parameters (Table 2) were taken to evaluate the influence of the treatment on the visual appearance of hydrocolloids. Similarly, the lightness value (L* > 95) and the whiteness index (WI > 90) combined are associated with the yellow color of the samples. Relative pH changes in the raw material process promote the browning of hydrocolloids when they are brought to alkaline conditions, lowering the levels of L and WI in all cases, except for the hydrocolloids extracted from the seed present grayish tones with values <70 in all pH treatments [55].

Regarding the color variation (Δ*E*), a decrease is observed as the pH of treatment (3–10) increases. The hydrocolloids of the peel (94.48 ± 0.76–88.7 ± 0.56); pulp (99.95 ± 0.14–67.57 ± 0.04); seed (67.67 ± 0.34–57.07 ± 0.44) show this trend. Otherwise, this characteristic is observed concerning microstructural properties. It is observed that the internal changes of the samples directly influence the visual effects since the size of the polymeric particles becomes more prominent with increasing pH, having greater absorbance capacities that reflect their color change, as they do not have reflection angles that scatter light.

### 2.2. Hydrocolloids Spectral Features

**Figure 2:**
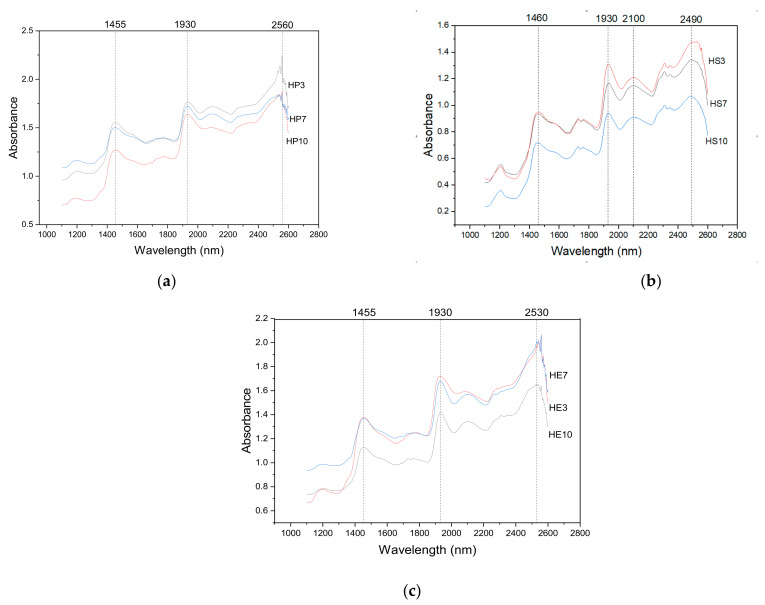
Representative NIR spectra of hydrocolloid; (**a**) Pulp; (**b**) Seed; (**c**). Peel.

The NIR spectra of the hydrocolloids are presented in Figure 2. The absorption intensity was significantly different between the seed, peels, and pulp samples, being the seed with the lowest absorbance degree between 0.2 and 0.5. The hydrocolloid from peel and pulp exhibited a higher amplitude and sharper peak depending on the ratio of the pH sample. The peak amplitude decreased as the pH variation increased; these differences in NIR spectral features reflect differences in water hydrocolloid interactions in the structure of gels [56,57,58,59]. As the NIR spectrum is sensitive to changes in hydrogen bonds and the packing of crystal cells, it can be detected. The polymorphic form or compound concentration can be carried out from a qualitative and quantitative point of view. In addition, for the wavelength, there is one major water band (O-H) discernable at around 1455–1460 nm and bands observable around 1930 nm, which represents the presence of carbohydrates (C-H) for all hydrocolloids [59,60]. The main components of plant tissue, which consist of various combinations of groups mentioned above, have absorption properties in this spectrum region [61].

Vibration differences in the range around 1300 to 1460 nm have been identified for fruits such as grape products, wine, citric, etc. [62,63,64], showing the vibration range of the c-H and OH peaks corresponding to water, combined with the absorbance of phenolic compounds. Moreover, the spectrum shows another absorbance peak, around 1950–2100 nm, in the hydrocolloid from the seed corresponding to protein groups (N-H) [61]. In the Pulp, the O-H group appears at 1455 nm and C-H at 1930 nm. In the seed, the O-H appears at 1460 nm, the C-H group appears at 1930, and N-H at 2100. Finally, Peel O-H appears at 1455 nm and C-H at 1930 nm. They are supported by the data obtained from the physiochemical characterization of the samples in Table 1.

### 2.3. Total Phenolic Compounds (TPC) and Antioxidant Activity

The total phenolic compounds and antioxidant activity of hydrocolloids are shown in Table 3. Hydrocolloids have TPC values in the range of 21.61 ± 0.39 to 51.77 ± 2.48 mg GAE/g, close to those reported by Sánchez-Camargo et al. [65] and Dorta et al. [66] in mango peel and seed. These phenolic compounds contribute significantly to the antioxidant activity of the hydrocolloids obtained, in accordance with studies in the scientific literature on phenolic compounds as antioxidants [67,68]. Furthermore, phenolic antioxidants are molecules that, in small concentrations, protect biomolecules (phospholipids, nucleic acids) from damage caused by free radicals [69,70]. The truth is that the nature of free radicals makes them very reactive [71]. However, phenolic compounds are capable, when colliding with them, of giving an electron or hydrogen, stabilizing, and thus preventing the initiation or oxidative damage propagation. Antioxidant activity measured through ABTS differed between the pulp, seed, and peel hydrocolloids, ranging between 152.38 and 198.85 TEAC (μMol Trolox/g); the ABTS radical scavenging capacity is equivalent to that of Trolox; therefore, higher values of μM of Trolox g^−1^ determines a higher antioxidant activity. In general, an increase in antioxidant activity is associated with high TPC. However, a correlation could be established with the treatments applied to materials with bioactive compounds, such as changes in temperature, pH, light intensity, and acidity [72].

However, for the ABTS radical test, the total antioxidant activity is equivalent to the concentration of the extract required to reduce the initial concentration of the ABTS radical by 50%; therefore, lower values correspond to higher antioxidant activity [73]. The values show a higher antioxidant activity of the hydrocolloids extracted from the peel and seed (≥193.82) and a lesser extent for the pulp. Similarly, hydrocolloids with these characteristics can be applied in different food matrices such as emulsions, sauces, and beverages, conferring added value to the final product [74,75,76,77,78]. The hydrocolloids obtained could then be used in the development of food products to reduce the oxidation process as antioxidants and preservatives.

### 2.4. Technological Properties

Variation in the mechanical properties of foods, such as texture, viscosity, and surface activity, gives rise to various physical properties that determine the functionality of hydrocolloids. Solubility is a factor of analysis that affects other functional properties and serves as a valuable performance indication of hydrocolloids in dispersion systems. Hydrocolloid interaction in aqueous systems reduces the diffusion and collision of suspended particles [79,80]. These must be adequately hydrated and solubilized to perform their function. They can be soluble in hot or cold water depending on factors such as the length of the polymeric chain and its ramifications, the way in which they are grouped, and the configuration of electrical charges [55].

#### 2.4.1. Solubility

**Figure 3:**
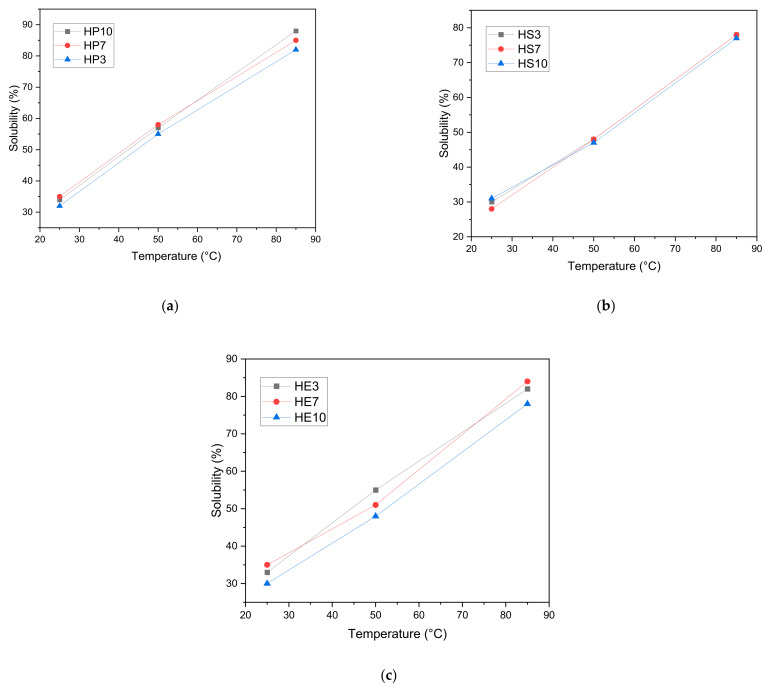
Sample solubility as a function of temperature from (**a**) Pulp; (**b**) Seed; (**c**). Peel.

Figure 3 presents the solubility properties of hydrocolloids from the pulp, seed, and peel of mango (*Mangifera indica*) var. hilaza. In general, they have an excellent solubility capacity. However, as the temperature increases, the ability to dissolve in water increases, similar property to that carried out by Sciarini et al. [81], where they evaluated the functional properties of *Gleditsia triacanthos* gum, having a maximum capacity of 80% solubility when exceeding 70 °C. Likewise, the study carried out by Salahi et al. [82] investigated the functional properties of a new gum from *Eremurus luteus* root. They presented similar solubility results between 50% and 80% when the temperature increases. These structural changes occur when breaking hydrogen (H) bonds between polysaccharide chains is favored, exposing OH groups that facilitate the ability to form H bonds with water. 

#### 2.4.2. Swelling Index (SI) and Water Holding Capacity (WHC)

**Figure 4:**
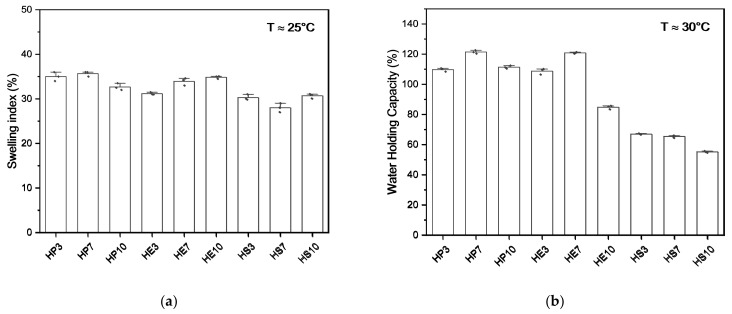
(**a**) Swelling index (SI) and (**b**) water holding capacity (WHC) of hydrocolloids from mango (*Mangifera indica*) var. hilaza.

The SI and WHC values are shown in Figure 4. The highest values were observed for hydrocolloids obtained from the pulp, followed by the peel and seed associated with their composition. Hydrocolloids from pulp and peel could retain more moisture due to CHO’s presence. When subjected to heating and changes in pH, they could cause the splitting of the constituent polysaccharides and their more significant proportion of branching [83]. These factors decreased the degree of chain-to-chain interaction. Therefore, it was easier for water to form bonds with polysaccharide chains; this may be explained because it increased molecular mobility, promoted water absorption, and increased the degree of water held by the hydrocolloids. Amid et al. [84] reported similar values when evaluating this property in green gram gum (139.5% WHC) and evenly in tamarind seed mucilage (107% WHC) at 60 °C, evaluated by Alpizar-Reyes et al. [4]. Then, seed hydrocolloids at pH 7 (HSph7) and pH 10 (HSph10) presented the lowest carbohydrate values and a higher proportion of protein chains, which require strong process conditions; these polymers interact strongly with each other and, to a lesser extent, with water. 

The swelling index (SI) indicates the degree of hydration of the granules. In this way, the increase in swelling capacity means weaker bonding forces; when the chains are separated, they increase the weaker bonding interactions between the polymer molecules. Therefore, the enlargement of molecules allows greater water retention. Additionally, the increase in the swelling index depends on the composition of the materials and the exposure of functional groups that can cause a greater volume available for the retention of water in the molecule. Furthermore, the swelling power index was correlated with WHC (the higher the swelling index, the higher water retention), with results between 28–35.2% SI. In that sense, the molecular weight of proteins is more significant than that of carbohydrates, representing an inversely proportional behavior (higher molecular weight-lower swelling capacity and water retention) [85].

### 2.5. Microstructural Characterization

**Figure 5:**
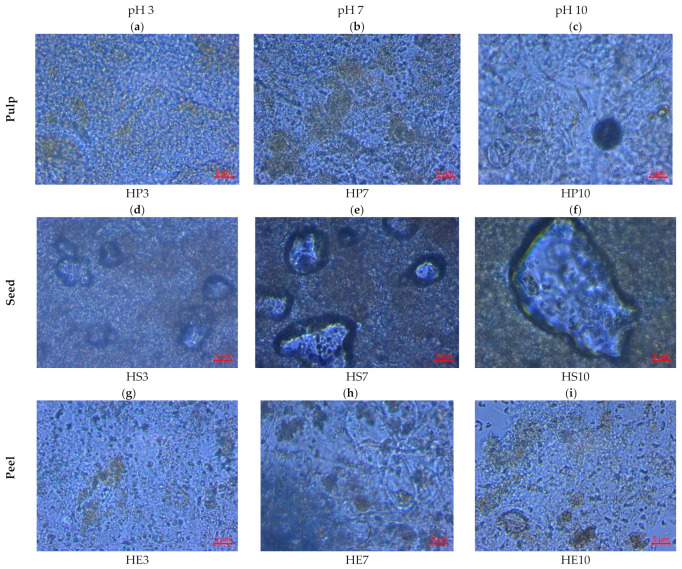
Microstructure of hydrocolloids from pulp, seed, and peel of mango (*Mangifera indica*) var. hilaza.

Figure 5 demonstrates the microstructural characteristics of the hydrocolloids obtained. The confocal microscope offers the possibility of analyzing transverse optical sections, allowing the internal sample structure to be observed without any special preparation, achieving it through the displacement of the focal plane through the sample, which is only possible to observe the model in the XY plane. Based on this, the hydrocolloid microstructural analysis shows dense areas and compact regions for hydrocolloids obtained from the pulp at pH 3 and 7 (Figure 5a,b). In seed, more compact regions were observed (Figure 5d–f), similar characteristics obtained by Lofgren et al. [86], where the structure of mixed pectin gels was studied and this polymeric network formed; being of great importance for the functional properties of solubility, swelling index, and water retention capacity. These results are according to the spectrum obtained from the NIR measurement, where characteristic peaks are observed exposing C-H and O-H groups that prove functional capacities such as solubility, swelling index, and water retention. This network that forms with the aqueous system can be observed in the pulp hydrocolloids obtained at pH 10 (Figure 5c), peel at pH 7 (Figure 5h), and seed, more clearly at pH 7 and 10 (Figure 5e,f) [82].

The results obtained at the microstructural level show that the extraction of hydrocolloids at different pH levels does not represent a differential parameter in the capacity to form a polymeric network, but if the aggregation state is attributed instead to its composition, as in the case of seed hydrocolloids where the protein chains (a major component in these samples) tend to be in the aggregation state, image (Figure 5d–f), which represents a higher molecular weight, linked with higher textural hardness and generally lower moisture content (Table 1) [85].

## 3. Conclusions

Hydrocolloids of the peel, pulp, and seed of mango (*Mangifera indica*) var. Hilaza were obtained at different pH treatments. The yields obtained have higher values when the raw material is solubilized at pH 3 or 10. Physicochemical analysis showed that the peel and pulp mainly have carbohydrates as a functional component and the seed has a higher protein content. However, besides having functional characteristics, hydrocolloids can provide phenolic compounds in food matrices with antioxidant capacity, representing an added value of nutritional traits. 

The microstructural characteristics showed that hydrocolloids obtained from the seed form compact regions and have the ability to form polymeric networks capable of retaining water as those obtained from the pula at pH 10 and the peel at pH 7. Color parameters are related to the extraction treatment of the hydrocolloids, where they vary Δ*E*, color variation. It is less than alkaline pH with brown tones and more yellow and white tones at acid pH conditions. NIR spectra are essential for validating the hydrocolloid composition, representing its degree of functionality and industrial applicability. They maintain a similar wavelength but differ in breadth, exposing the O-H, C-H, and N-H groups and modifying the solution conditions and functional properties. Therefore, the hydrocolloids obtained are a new alternative for the exploitation of mango at the industrial level and can be used in food matrices such as jams, juices, sauces, etc.; with functional (solubility, swelling, and water holding properties) and biological benefits (antioxidant capacity whit phenolic compounds) properties.

## 4. Materials and Methods

### 4.1. Chemical Reagents

Ethanol (Analytical grade, 99.5%) and glacial acetic acid (99.5%) were purchased from Panreac (Bacelona-Spain); sodium hydroxide (99.5%) from EMSURE (Darmstadt Germany), anhydrous sodium carbonate (99.5%), gallic acid standard (>98%), phenylmethyl siloxane (5%), Folin–Ciocalteu reagent, 2,2′-azino-bis (3-ethylbenzothiazoline-6-sulfonic acid) diammonium salt (ABTS, ≥95%) were obtained from Sigma Aldrich (St. Louis, MO, USA).

### 4.2. Materials

Mango (*Mangifera indica*) var. Hilaza was purchased from the local market of Cartagena de Indias (Colombia) in a state of organoleptic maturity. The whole fruits were sanitized by immersion in a sodium hypochlorite solution (100 mg/L) for 10 min at a temperature of 30 °C. Peel, pulp, and seed were manually separated with stainless steel knives. Subsequently, the pulp, seed, and peel were lyophilized for 72 h using Labconco Freezone 1.5 L benchtop equipment. Subsequently, a reduction in particle size was made in an IKA MF 10.2 mill coupled with a sieve to obtain particle sizes smaller than 250 µm.

### 4.3. Hydrocolloid Extraction

Hydrocolloid extraction was carried out following the procedures described by Quintana et al. [87] and López-Barraza et al. [7] with some modifications, evaluating the parts of mango (pulp, peel, and seed) and three pH (3, 7, and 10) of solubilization obtaining nine experiments (Table 4). Briefly, the material (part of mango) was solubilized with distilled water in a 1:8 ratio followed by adjustment of pH with acetic acid (1 N) and NaOH (0.1 N) continuously stirred for 4 h at 80 °C and filtered. The solubilized mixture was then mixed with ethanol (1:1 ratio) at 0 °C to favor hydrocolloid precipitation (3 h). The mixture was centrifuged for 15 min at 4000 rpm, and the sediment was lyophilized and milled to obtain dry hydrocolloid.

The extraction yield was calculated considering the amount of dry raw material used and the amount of product obtained after the extraction process using Equation (1).

(1) $$\%\;\mathrm{yield}=\frac{\mathrm{the}\;\mathrm{weight}\;\mathrm{of}\;\mathrm{dried}\;\mathrm{hydrocolloid}\;\mathrm{obtained}}{\mathrm{weight}\;\mathrm{from}\;\mathrm{raw}\;\mathrm{material}}\times 100$$

### 4.4. Physicochemical and Proximal Analysis

Moisture, ether extract, ash, and protein content of hydrocolloids were determined using standard methods of Association of Official Analytical Chemists- AOAC No. 926.08, 972.28, 935.42, and 926.123, respectively. The carbohydrate content was determined by the difference [88]. 

Titratable acidity was determined based on alkalimetric titration with 0.1 N NaOH using phenolphthalein as an indicator (AOAC No.967.21). The pH was measured using a Metter Toledo AG SG2 digital potentiometer, previously calibrated, according to Method 942.05 described by the AOAC [88].

Hydrocolloid color was determined using the method described by Shittu et al. [89]. The histogram of light-dark (L*), greenish/red (a), and bluish yellow (b) allows one to calculate the whiteness index (WI) and the color variation (Δ*E*) by Equations (2) and (3), respectively [89,90,91].

(2) $$\mathrm{WI}=100-{\left[{\left(100-L^{*}\right)}^{2}+a^{2}+b^{2}\right]}^{0.5}$$

(3) $$\Delta E={\left[{\left({\Delta L}^{*}\right)}^{2}+{\left(\Delta a\right)}^{2}+{\left(\Delta b\right)}^{2}\right]}^{0.5}$$

### 4.5. NIR Spectral Measurement

NIR spectral measurements were made following the procedures described by Huang et al. [59] and Lundin et al. [57] using a SpectraStar^TM^ XT NIR analyzer Series with scanning monochromator technology, an operating range of 1100–2600 nm, and spectral resolutions of 2.5 nm; equipment with Si and Pbs detectors. To analyze the organic functional groups (especially O-H, N-H, and C=O), the absorbance data were determined. Each measurement was preceded and followed by calibration scans against air 1.9 scan/s. The diameter of the fiber optic probe was approximately 5 cm; for spectral collection, the probe was placed in direct contact with the surface of dry hydrocolloids.

### 4.6. Determination of Total Phenolic Compounds

The total phenolic compounds of the hydrocolloids obtained were determined using the Folin-Ciocalteu singleton method [92]. Briefly, 50 mg of hydrocolloid are mixed with 3 mL of MilliQ water and 250 mL of Folin Ciocalteu reagent. The contents were thoroughly mixed, and after 3 min, 750 mL of sodium carbonate solution and 950 mL of distilled water were added. The mixture is kept for two hours at room temperature and in the dark, then the absorbance measurement at 760 nm will be performed using a Genesys 10S UV-Vis spectrophotometer (Thermo Fischer Scientific Inc., Miami, FL, USA). The results will be expressed as GAE (mg of gallic acid equivalents/g of extract). All analyzes will be performed in triplicate.

### 4.7. Determination of Antioxidant Activity

The antioxidant capacity of the extracts was determined by the ABTS + radical scavenging assay following the method described by Re et al. [93] ABTS + radical cation of ABTS + was generated by mixing ABTS + stock solution of ABTS + (7 mM) with 2.45 mM potassium persulfate after incubation of the mixture at room temperature for 16 h in darkness. Once the ABTS + radical was formed, the solution absorbance was adjusted to 0.700 ± 0.02 at 734 nm by ethanol in a Genesys 10S UV–vis spectrophotometer (Thermo Fischer Scientific Inc., Miami, FL, USA). Afterward, 990 μL of ABTS + solution was added to 10 μL of the sample, and the reaction mixture was allowed to stand at room temperature and under darkness until the absorbance reached a plateau. The absorbance was recorded at 734 nm, and the results were expressed as the value IC_50_ value (Inhibitory concentration: extract concentration necessary to inhibit the initial concentration of radical by 50%), as well as equivalent Trolox (TEAC) (μmol Trolox/g extract), which were calculated taking into account the Trolox standard and sample concentrations that produce the elimination of 50% of ABTS + radical. All analyzes were performed in triplicate.

### 4.8. Techno-Functional Properties

#### 4.8.1. Solubility

The solubility (%*Solubility*) of hydrocolloids was measured using the method described by Sciarini [81] with some modifications. Accordingly, 500 mg of hydrocolloids were mixed with 50 mL of water at 25, 65, and 90 °C for 35 min and cooled at 25 °C. After that, the mixture was centrifuged at 3000 rpm for 15 min. The supernatant was separated and dried at 110 °C for 24 h. The %*Solubility* was calculated using Equation (4).

(4) $$\%\mathrm{Solubility}=\frac{\mathrm{Dry}\;\mathrm{weight}}{\mathrm{Sample}\;\mathrm{weight}}\times 100$$

#### 4.8.2. Swelling Index (%SI)

The swelling index (%SI) was determined following the procedures described by Kalegowda et al. [94] and Archara et al. [95] with some modifications. Briefly, 500 mg of hydrocolloid were placed in a 10 mL graduated test tube. Further, 2 mL of alcohol (95%) was added to reach a good dispersion; after that, distilled water was added to a distribution of 10 mL. The solution that became viscous was stored at room temperature, and the final volume of the sediment was recorded after 24 h. The swelling index was calculated by determining the relationship between the swollen volume and the initial volume using Equation (5).

(5) $$\%\mathrm{SI}=\frac{\mathrm{Initial}\;\mathrm{volume}}{\mathrm{Final}\;\mathrm{volume}}\times 100$$

#### 4.8.3. Water Holding Capacity (%WCH)

The water holding capacity (%WCH) was determined following the procedures described by Ghribi et al. [96]. Accordingly, 500 mg of hydrocolloids was placed in a centrifuge tube, adding an excess of 3 mL of water and shaking for one minute. Subsequently, the tubes were centrifuged at 3200 rpm for 30 min at 30 °C to measure the volume of water that was not retained. The amount of water retention was expressed using Equation (6).

(6) $$\%\mathrm{WCH}=\frac{\mathrm{water}\;\mathrm{absorbed}\;\mathrm{volume}}{\mathrm{sample}\;\mathrm{weight}}\times 100$$

### 4.9. Microstructural Analysis

For structural analysis, a primo Star optical microscope (Carl Zeiss Primo Star Microscopy GmbH, Jena, Germany) was set up using a 100× magnification lens to observe the internal distribution of wet hydrocolloids extracted from MH. The equipment has a built-in digital camera (DCMC310) with Scope Photo software (version 3.1.616) from Hangzhou Huaxin Digital Technology Co., Ltd., Zhejiang, China, which allowed snapshots to be taken as the observation was made.

### 4.10. Statistical Analysis

Data were analyzed by ANOVA (unidirectional) with Tukey’s HSD (honestly-significant difference) test, using SPSS software (version 17.2 for Windows) to determine statistically significant differences (*p* < 0.05) between samples. All tests were performed in triplicate.

## Figures and Tables

**Table 1:** Proximal composition and physicochemical properties of the hydrocolloids of mango (*Mangifera indica*) var. hilaza. at different pH.

Sample Code	pH *	SS * Brix	Acidity %	Moisture %	Ash %	Lipids %	Carbohydrate %	Proteins %
HP3	3.51 ^ac^	10.56 ^ab^	0.17 ± 0.78 ^c^	75.38 ± 0.98 ^bc^	1.61 ± 0.54 ^abd^	0.63 ± 0.12 ^ac^	21.59 ± 2.11 ^ab^	0.33 ± 0.03 ^abc^
HP7	6.73 ^abc^	10.45 ^abc^	0.06 ± 0.99 ^ac^	74.25 ± 1.43 ^bd^	0.48 ± 0.36 ^ac^	0.65 ± 1.04 ^abc^	23.52 ± 1.03 ^abc^	0.38 ± 0.02 ^ac^
HP10	8.15 ^bc^	10.58 ^abc^	0.01 ± 0.41 ^abc^	72.10 ± 2.35 ^abc^	0.94 ± 0.63 ^abe^	0.85 ± 0.50 ^abe^	23.06 ± 1.70 ^ac^	0.42 ± 0.09 ^bc^
HE3	4.54 ^adc^	4.21 ^acd^	0.17 ± 0.48 ^adc^	75.32 ± 1.21 ^ad^	1.30 ± 0.61 ^abc^	0.93 ± 0.01 ^abc^	21.20 ± 1.20 ^abc^	0.35 ± 0.03 ^abc^
HE7	6.78 ^ab^	4.35 ^bc^	0.08 ± 0.05 ^abd^	74.13± 0.95 ^ab^	1.52 ± 0.48 ^ab^	0.87 ± 0.25 ^abc^	22.45 ± 1.08 ^ac^	0.41 ± 0.08 ^abe^
HE10	8.72 ^ad^	4.65 ^ac^	0.01 ± 0.06 ^ab^	74.52 ± 1.43 ^acd^	1.10 ± 0.71 ^abc^	0.78 ± 0.31 ^ab^	22.12 ± 0.54 ^bc^	0.37 ± 0.03 ^abe^
HS3	4.25 ^abc^	2.56 ^abc^	0.17 ± 0.58 ^bc^	65.74 ± 0.23 ^bd^	1.47 ± 0.46 ^abd^	9.86 ± 1.35 ^ac^	2.06 ± 0.30 ^abc^	20.24 ± 0.96 ^acd^
HS7	6.54 ^bc^	2.34 ^ab^	0.05 ± 0.05 ^abc^	63.07 ± 1.44 ^ad^	1.32 ± 0.85 ^ab^	10.32 ± 1.73 ^ad^	2.32 ± 0.40 ^ac^	20.93 ± 1.21 ^ac^
HS10	8.21 ^abc^	3.12 ^abd^	0.02 ± 0.11 ^cd^	62.96 ± 0.83 ^ab^	1.65 ± 0.53 ^ac^	11.15 ± 0.98 ^ad^	2.63 ± 0.90 ^abc^	20.36 ± 0.67 ^dc^

The proximal compositions were determined on a wet basis, which is not reported in the literature or reported on a wet basis. Data are expressed as mean ± standard deviation. Different letters in the same column express statistically significant differences (*p* < 0.05). * pH and soluble solids (SS) have a coefficient of variation of <0.05.

**Table 2:** Color parameters of hydrocolloids extracted from mango (*Mangifera indica*) var. hilaza. at different pH.

Sample Code	L*	WI	Δ*E*
HP3	94.46 ± 0.56 ^c^	94.15 ± 0.45 ^a^	94.48 ± 0.76 ^cb^
HP7	97.07 ± 0.18 ^bc^	95.78 ± 0.29 ^cb^	97.11 ± 0.64 ^ac^
HP10	88.20 ± 0.58 ^b^	87.75 ± 0.65 ^abc^	88.7 ± 0.56 ^ab^
HS3	67.47 ± 1.14 ^abc^	67.05 ± 0.90 ^b^	67.67 ± 0.34 ^a^
HS7	57.96± 0.85 ^cb^	57.78± 0.81 ^bc^	58.09 ± 0.72 ^a^
HS10	56.87± 0.45 ^abc^	56.62 ± 0.36 ^c^	57.07 ± 0.44 ^b^
HE3	98.05 ± 0.14 ^bc^	80.49 ± 0.42 ^bc^	99.95 ± 0.14 ^c^
HE7	67.38 ± 1.45 ^a^	66.22 ± 0.35 ^ac^	67.98 ± 0.42 ^ac^
HE10	67.45 ± 1.12 ^abc^	67.23 ± 0.85 ^cb^	67.57 ± 0.04 ^b^

L*, lightness; WI, whiteness index; Δ*E*, color variation. Data are expressed as mean ± standard deviation. Different letters in the same column express statistically significant differences for hydrocolloid extraction at pH 3, 7, and 10 (*p* < 0.05). pH and soluble solids (SS) have a coefficient of variation of <0.05.

**Table 3:** The total phenolic compounds (TPC) and antioxidant activity (IC_50_ and TEAC) of the hydrocolloids were obtained from mango (*Mangifera indica*) var. hilaza. at different pH.

Sample Code	TPC mg GAE/g	IC_50_ μL/g	TEAC μMol Trolox/g
HP3	31.69 ± 4.50 ^bc^	49.98 ± 0.22 ^a^	152.38 ± 0.66 ^c^
HP7	25.17 ± 0.18 ^abc^	46.36 ± 0.07 ^d^	164.29 ± 0.24 ^a^
HP10	32.13 ± 2.17 ^ab^	41.67 ± 0.05 ^abc^	185.03 ± 0.23 ^a^
HS3	47.98 ± 2.17 ^abc^	38.84 ± 0.19 ^d^	196.44 ± 0.95 ^a^
HS7	46.61 ± 0.85 ^c^	36.77 ± 0.78 ^abc^	198.73 ± 0.72 ^a^
HS10	21.61 ± 0.39 ^abc^	43.83 ± 0.34 ^d^	193.82 ± 1.34 ^a^
HE3	37.34 ± 1.16 ^abc^	38.23 ± 0.12 ^d^	198.85 ± 0.76 ^a^
HE7	51.77 ± 2.48 ^a^	37.25 ± 0.93 ^abc^	197.55 ± 0.02 ^a^
HE10	28.68 ± 4.50 ^abc^	38.16 ± 0.07	196.98 ± 0.37 ^c^

Data are expressed as mean ± standard deviation. Different letters in the same column express statistically significant differences for hydrocolloid extraction at pH 3, 7, and 10 (*p* < 0.05).

**Table 4:** Extraction conditions of hydrocolloids from pulp, peel, and seed from mango (*Mangifera indica*) var. hilaza.

Sample Code	Parts of Mango	pH	Temperature °C	Time Hours
HP3	Pulp	3	80	4
HP7	Pulp	7	80	4
HP10	Pulp	10	80	4
HE3	Peel	3	80	4
HE7	Peel	7	80	4
HE10	Peel	10	80	4
HS3	Seed	3	80	4
HS7	Seed	7	80	4
H10	Seed	10	80	4

## Data Availability

Not applicable.
